# Discovering metabolic disease gene interactions by correlated effects on cellular morphology

**DOI:** 10.1016/j.molmet.2019.03.001

**Published:** 2019-03-13

**Authors:** Yang Jiao, Umer Ahmed, M.F. Michelle Sim, Andrea Bejar, Xiaolan Zhang, M. Mesbah Uddin Talukder, Robert Rice, Jason Flannick, Anna I. Podgornaia, Dermot F. Reilly, Jesse M. Engreitz, Maria Kost-Alimova, Kate Hartland, Josep-Maria Mercader, Sara Georges, Vilas Wagh, Marija Tadin-Strapps, John G. Doench, J. Michael Edwardson, Justin J. Rochford, Evan D. Rosen, Amit R. Majithia

**Affiliations:** 1 Broad Institute of MIT and Harvard, Cambridge, MA 02142, USA; 2 University of Cambridge Metabolic Research Laboratories, Institute of Metabolic Science, Addenbrooke's Hospital, Cambridge, CB2 0QQ, UK; 3 Department of Pharmacology, University of Cambridge, Cambridge CB2 1PD, UK; 4 Genetics and Pharmacogenomics, Merck & Co., Inc., Boston, MA 02115, USA; 5 Rowett Institute and the Aberdeen Cardiovascular and Diabetes Centre, University of Aberdeen, Foresterhill, Aberdeen AB25 2ZD, UK; 6 Division of Endocrinology, Diabetes and Obesity, Beth Israel Deaconess Medical Center, Boston, MA 02215, USA; 7 Harvard Medical School, Department of Genetics, Boston, MA 02215, USA; 8 Division of Endocrinology, Department of Medicine, University of California San Diego, La Jolla, CA 92093, USA

**Keywords:** Gene discovery, Functional genomics, Metabolic syndrome, Insulin resistance, Type 2 diabetes, Genetic screen, High content imaging, Lipodystrophy

## Abstract

**Objective:**

Impaired expansion of peripheral fat contributes to the pathogenesis of insulin resistance and Type 2 Diabetes (T2D). We aimed to identify novel disease–gene interactions during adipocyte differentiation.

**Methods:**

Genes in disease-associated loci for T2D, adiposity and insulin resistance were ranked according to expression in human adipocytes. The top 125 genes were ablated in human pre-adipocytes via CRISPR/CAS9 and the resulting cellular phenotypes quantified during adipocyte differentiation with high-content microscopy and automated image analysis. Morphometric measurements were extracted from all images and used to construct morphologic profiles for each gene.

**Results:**

Over 10^7^ morphometric measurements were obtained. Clustering of the morphologic profiles accross all genes revealed a group of 14 genes characterized by decreased lipid accumulation, and enriched for known lipodystrophy genes. For two lipodystrophy genes, BSCL2 and AGPAT2, sub-clusters with PLIN1 and CEBPA identifed by morphological similarity were validated by independent experiments as novel protein–protein and gene regulatory interactions.

**Conclusions:**

A morphometric approach in adipocytes can resolve multiple cellular mechanisms for metabolic disease loci; this approach enables mechanistic interrogation of the hundreds of metabolic disease loci whose function still remains unknown.

## Highlights

• Loss-of-function genetic screen in human adipocytes for 125 genes selected from metabolic disease-associated loci.
• Genetic screen read out by cellular morphometry— 77,000 images taken with 400 morphological features extracted per image.
• Pairwise mechanistic interactions between genes identified by correlations of cellular morphometry—two interactions validated.
• Novel interaction between BSCL2 and PLIN1 from biophysical association of proteins at lipid droplet surface.
• Novel interaction between CEBPA and AGPAT2 from CEBPA dependent transcription of AGPAT2.

## 1 Introduction

Modern genome engineering methods now make it feasible to selectively inactivate any gene in any cultured human cell [1]. In the past decade, a plethora of disease relevant genetic loci have been identified by genetic association studies (e.g. GWAS) of clinically phenotyped populations for metabolic diseases/traits including Type 2 Diabetes, waist-to-hip ratio, body mass index and fasting insulin [2]. These advances have the potential to accelerate therapeutic pathway discovery by enabling the perturbation of many disease genes in parallel in any cell model. What is now needed are scalable readouts that can interrogate multiple mechanisms and disease relevant cell models in which to apply them. Multiparametric “omic” readouts quantifying general processes such as gene transcription have proven scalable and well-suited to interrogating a variety of molecular mechanisms [3].

Cellular morphology, i.e. how a cell appears under the microscope, is a potentially even more general readout as it represents the amalgamation of genetic, transcriptional, and proteomic states [4], [5]. Cellular morphology has been successfully deployed to interrogate subcellular mechanisms [6] and several recent studies have demonstrated the feasibility of combining morphological readout with gain/loss-of-function genetic perturbations to functionally annotate genes into molecular pathways [7], [8]. Thus far, however, the mechanistic information gained has been limited to cell growth, mitosis and proliferation, likely due to the use of heterologous and cancer cell models which are not suitable for studying most phenotypes of interest.

Most adult onset genetic diseases do not manifest in every cell in the body [9] making it likely that the study of disease loci/genes in cell types matched to disease is more likely to yield relevant cellular mechanisms. As a case in point, familial [10] and population [11], [12], [13], [14] based genetic studies of metabolic disease have identified dozens of genes expressed in adipocytes which confer risk by altering cellular features in adipocytes. The functional pathways altered in these systems remain poorly characterized.

We hypothesized that perturbing these loci/genes in adipocytes *in vitro* and assessing the effect on morphologic features would enable disease relevant functional annotation and yield diverse mechanistic insights regarding insulin resistance and adipocyte differentiation. Here, we demonstrate the utility of this approach in metabolic disease. We selected 125 genes by filtering associated loci from metabolic disease association catalogs for adipocyte expression, and then ablated these genes in human pre-adipocytes using CRISPR/CAS9. We then profiled the effect on cellular morphology using morphologic similarity to identify mechanistic interactions between genes. We demonstrate that our morphometric approach is capable of surveying diverse cellular mechanisms by validating both a protein–protein interaction on the lipid droplet surface and a transcriptional regulatory interaction in the DNA.

## 2 Methods

### 2.1 Lentiviral gene ablation constructs

For the each of the 133 selected genes (125 metabolic disease genes and controls and 8 essential gene controls) (Supplementary Table 1), three CRISPR/CAS9 constructs were designed using “Ruleset 2” as described previously [15] and instantiated in (https://portals.broadinstitute.org/gpp/public/analysis-tools/sgrna-design). The designed constructs were cloned into a lentiviral transduction vector (lentiCRISPRv2) which contained a CAS9 transgene and a mammalian antibiotic resistance cassette for puromycin. An additional 25 distinct constructs targeting no genomic sequence (non-targeting controls) were cloned. Lentivirus was produced from the resulting construct array using standard protocols (https://portals.broadinstitute.org/gpp/public/resources/protocols).

### 2.2 Genetic ablation, mutation quantification and imaging in SGBS adipocytes

**Figure 1:**
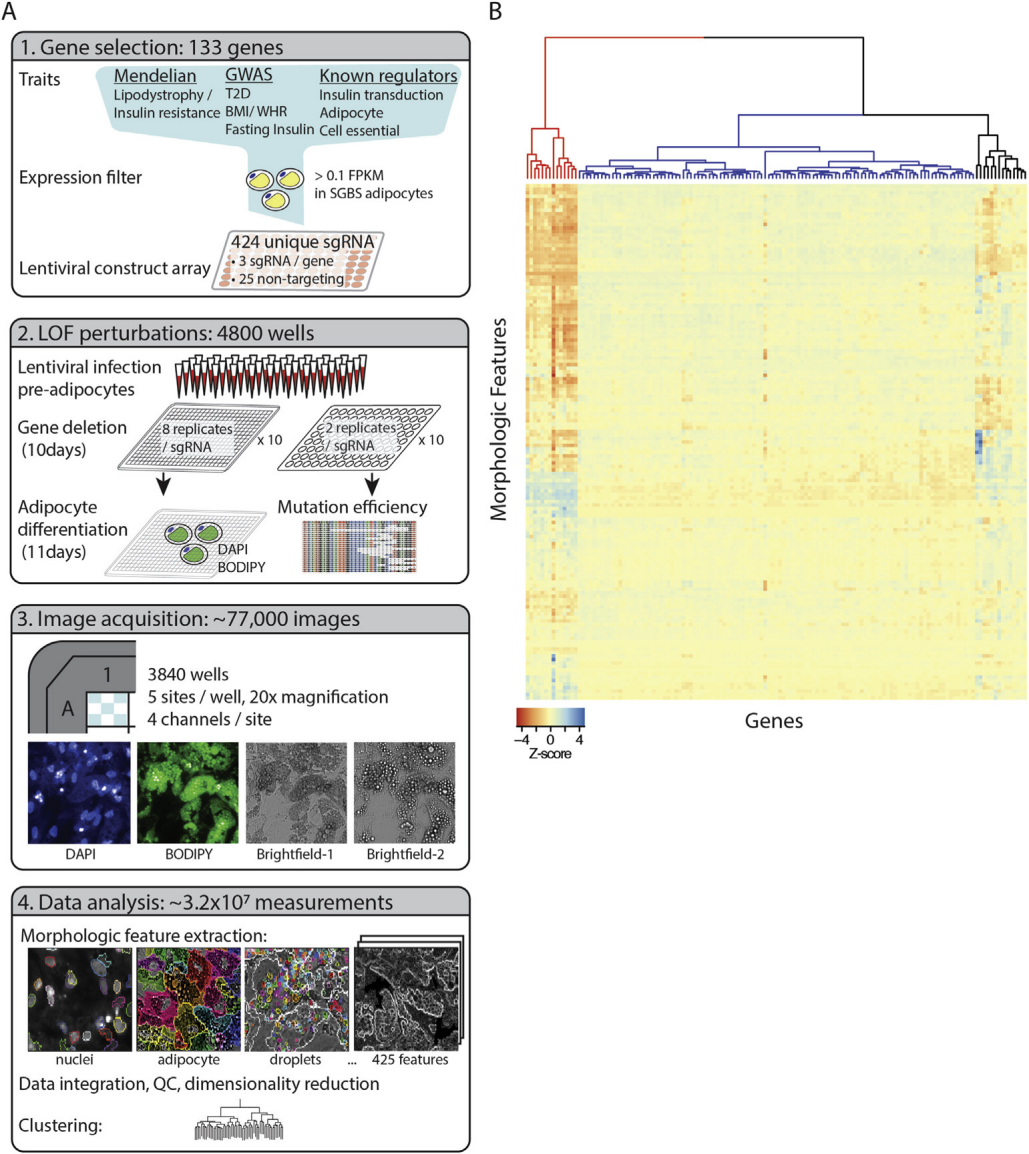
**Functional interaction of metabolic disease-associated loci in human adipocytes by morphologic profiling.** (A) [1] Genes were systematically identified from both Mendelian and common forms of metabolic disease alongside known regulators of adipocyte function and insulin signaling. This list was filtered by gene expression in differentiating SGBS cells resulting in 125 genes for study. Three independent CRISPR/CAS9 constructs were designed for each gene alongside non-targeting controls, and cloned for arrayed lentiviral transduction. [2] SGBS pre-adipocytes were infected with the lentiviral guide array and incubated for 10 days to allow gene knockout. After incubation transduced cells were differentiated into adipocytes; selected constructs were re-infected for assessment of gene modification/mutation efficiency. [3] Differentiated cells were stained for lipid (BODIPY) and nuclei (DAPI) and imaged in the corresponding fluorescent channels along with two types of brightfield images on an automated high-content microscope (Opera Phenix). [4] Each image was processed to segment cells, extract and numerically quantify 425 morphologic features. The resulting data were filtered for quality, removing poorly reproducible and redundant features to create morphologic profiles for each of the genes studied. The morphologic features were clustered to relate genes to one another and obtain novel mechanistic insight. (B) Morphologic profiles of 125 metabolic disease genes and 8 essential gene controls perturbed in human adipocytes. *Z*-scores of the 425 morphologic features (rows) for the 133 perturbed genes (columns) are displayed as a heatmap. The genes are clustered by morphologic similarity among the gene knockouts. The three major branches of the clustering dendrogram are color-coded as described in detail in the methods and Figure 2.

The lentiviral guide array described above was transduced into SGBS pre-adipocytes (gift from Martin Wabitsch) as previously described in details [16]. In brief, for assessment of targeting efficiency SGBS pre-adipocytes were plated in 96-well plates at 5000 cell/cm^2^ with 2 biological replicates per targeting construct, selected with puromycin and incubated for ten days prior to extraction of genomic DNA, PCR and shotgun sequencing by standard protocols (https://portals.broadinstitute.org/gpp/public/resources/protocols). Gene modification efficiency from the resulting sequences using CrisprVariants software with default parameters [17] (Supplementary Table 1). For imaging and morphologic profiling experiments (Figure 1A), the lentiviral array was transduced into SGBS pre-adipocytes plated at two densities (5000 and 8000 cells/cm^2^) and with four biological replicates per targeting construct at each density. The plate position for the biological replicates for each targeting construct was permuted so as to randomize potential systematic confounders such as plate position and seeding density. Infected cells were selected with puromycin, incubated for 10 days and then stimulated to differentiate under standard adipogenic condition [18]. Following differentiation, cells were fixed and stained for nuclei (DAPI) and lipid (BODIPY) as previously described [16].

The fixed and stained cells were imaged on a high-content imaging platform (Opera Phenix™) at 20× magnification in three channels corresponding to DAPI, BODIPY, and brightfield ×2. Each well was imaged at five sites (Figure 1A).

### 2.3 Image processing, filtering, feature aggregation and data analysis

Morphologic features were extracted from each cell using the Harmony™ high-content image analysis software. The DAPI channel was utilized to identify nuclei, extract related parameters, and the residual cytoplasmic DAPI staining to segment cells. The BODIPY and brightfield channels were used to identify lipid droplets and extract related intensity, morphology and texture parameters.

All features were measured per cell and summarized (mean and standard deviation) for each well resulting in a total of 425 summarized features for each well (Supplementary Table 2) using the Harmony software. Subsequent analyses were done in R3.42 using base packages unless noted. First features were filtered for reproducibility by computing the six possible pairwise Pearson correlation coefficient combinations among the four biological replicates for each guide; features with a negative correlation coefficient or two-sided *p* > 0.05 were removed (Supplementary Table 2: “non-reproducible”). Subsequently, each feature was *Z*-normalized across all guides and filtered for redundancy by pruning features that exceeded a Pearson *r* > 0.9 in the correlation matrix computed across all features (Supplementary Table 2: “correlated”; caret package [19]).

Subsequently for each feature, the *Z*-values across all guides for a given gene were averaged (median) to produce a table of 133 gene × 148 feature matrix for clustering (Figure 1B). Clustering was performed on euclidean distance matrix using Ward's method [20] (“Ward.D2” in R3.42). *p*-Values for clusters were calculated using multiscale bootstrap resampling as instantiated in R [21] (pvclust package: 10,000 bootstrap replications; Supplementary Figure 1).

### 2.4 Seipin and perilipin transgene constructs

Constructs to express FLAG-seipin and FLAG-perilipin were generated in the pCMV3xFLAG vector (Sigma Aldrich). Seipin-Myc and perilipin-Myc were in the pcDNA3.1 MycHis vector (LifeTechnologies). Fusion constructs for BiFC experiments were generated by inserting the N-terminal (1–158) fragment of yellow fluorescent protein (YFP) either downstream or upstream of seipin in pCMV3xFLAG to generate S–Yn and Yn–S fusion constructs, respectively, as previously described [22]. The C-terminal (155–239) fragment of YFP was amplified and inserted downstream or upstream of perilipin in pcDNA3.1. Wild-type Myc-seipin (WT) or mutants lacking the N terminus, first transmembrane domain, ER luminal loop region, second transmembrane domain, or the C terminus were a gift from Daisuke Ito, Keio University, Japan [23].

### 2.5 Co-IP of perilipin with seipin in human adipocytes

pcDNA3.1 Seipin-Myc (LifeTechnologies) was subcloned into pLXI_TRC401, a lentiviral expression vector containing an inducible Tet-on TRE promoter, by restriction cloning using NheI and BsrGI. The resulting pLXI-Seipin-MycHis construct contained the Seipin cDNA with a C-terminal MycHis tag and a subsequent stop codon. SGBS pre-adipocytes were transduced with pLXI-Seipin-MycHis lentiviruses and selected with puromycin as previously described [16]. Following selection, the transduced pre-adipocytes were grown to confluence and stimulated with an adipocyte differentiation cocktail [18] as well as doxycycline to stimulate seipin transgene expression. After seven days, the partially differentiated cells were collected for Co-IP. About 50,000 cells were lysed in 200 μl Pierce™ IP Lysis Buffer (Thermo Fisher 87788) plus Halt™ Protease and Phosphatase Inhibitor Cocktail (100×) (Thermo Fisher 78440). Lysates were rotated at 4 °C for 30 min and then centrifuged at maximum speed for 30 min at 4 °C. Supernatants were collected and protein concentration was determined using Micro BCA™ Protein Assay Kit (Thermo Fisher 23235). Perilipin was pulled down using anti-perilipin XP Rabbit mAb (CST 9349) with a Protein A bead slurry (CST 9863). Bound beads were then pelleted, washed and heated for 5 min at 95 °C. Immunoprecipitated samples were electrophoresed using Mini Protein TGX gels (Bio Rad 4561095) for 30 min at 200 V and transferred to nitrocellulose membranes. Membranes were blocked in 5% milk (in TBST) for 1 h and then incubated overnight at 4 °C with anti-Perilipin (1:1000) (CST 9349), anti-Seipin (1:5000) (CST 23846) antibodies in 5% BSA, anti-c-Myc (1:10,000) (Novus NB600-335) and anti-Cyclophilin A (1:1000) (Abcam ab58144) antibodies in 1% non-fat milk. Membranes were subsequently incubated in Clean Blot secondary antibody (1:400 in 5% non-fat milk in TBST), anti-c-Myc blots in anti-goat secondary antibody (1:2000 in 2.5% non-fat milk in TBST) (R&D HAF109), and cyclophilin A blots with anti-mouse secondary (1:2000 in 5% non-fat milk in TBST) (CST 7076) for 1 h. Blots were developed using Pierce ECL Western Blot Substrate (Thermo Fisher 32106).

### 2.6 Bimolecular fluorescence complementation (BiFC) analysis

3T3-L1 cells were seeded and allowed to grow to confluence (day −2), fed with fresh medium for two further days, before being differentiated on day 0. Cells were transfected with the desired BiFC constructs using Lipofectamine LTX reagent on day 2 of differentiation then incubated at 37 °C for 48 h. This was followed by a shift to 32 °C for 20 h on day 4 of differentiation, then to 30 °C for 4 h on day 5, before fixation for analysis by confocal microscopy. Cells were immunostained with anti-FLAG (seipin) or anti-Myc (perilipin) antibodies as described previously [22].

### 2.7 Atomic force microscopy

AFM experiments were performed as previously described in detail [22], [24]. Briefly, tsA 201 cells (a subclone of HEK293) were transiently transfected with a total of 250 mg of DNA in 5 × 162 cm^2^ culture flasks using calcium phosphate precipitation. Cells were harvested 48 h later, lysed and proteins isolated and eluted from anti-Myc (seipin) agarose. Perilipin in the eluates was detected on blots using an antibody to its FLAG tag. Isolated proteins were imaged using ‘tapping’ mode in air. Particle heights and diameters were measured manually by the Nanoscope software and used to calculate the molecular volume of each particle using the equation

(1) $$V_{\text{m}}=\left(\pi h/6\right)\left(3r^{2}+h^{2}\right)$$

where *h* is the particle height and *r* is the radius. Molecular volume based on molecular mass was calculated using the equation

(2) $$V_{\text{c}}=\left(M_{0}/N_{0}\right)\left(V_{1}+dV_{2}\right)$$

where *M*_0_ is the molecular mass, *N*_0_ is Avogadro's number, *V*_1_ and *V*_2_ are the partial specific volumes of particle (0.74 cm^3^/g) and water (1 cm^3^/g), respectively, and *d* is the extent of protein hydration (taken as 0.4 g water/g protein).

### 2.8 Immunoprecipitation of perilipin and deletion mutants of seipin

HEK293 cells were transfected with FLAG-perilipin in the absence or presence of either wild-type or mutant forms of Myc-tagged seipin. Lysates and anti-FLAG immunoprecipitates were prepared as described previously [22]. Forty-eight hours after transfection HEK293 cells were lysed in buffer comprising 50 mM n-octyl-b-d-glucopyranoside, 50 mM Tris, pH 6.8, 150 mM NaCl, 1 mM EDTA supplemented with protease inhibitors (Complete EDTA-free, Roche Applied Science) and phosphatase inhibitor cocktails (Sigma). Cells were sonicated, centrifuged at 16,000 g for 10 min at 4 °C. Lysate containing 1 mg of protein was added to 30 μl of anti-FLAG-agarose beads (Sigma–Aldrich) pre-equilibrated with lysis buffer and rotated gently for 2 h at 4 °C. Following centrifugation (8200 g for 30 s at 4 °C) supernatants were removed and beads washed three times with lysis buffer. FLAG-tagged proteins were eluted from beads by addition of 100 μl of 200 ng/μl 3 × FLAG peptide (Sigma–Aldrich) in TBS (50 mM Tris–HCl, pH 7.4, 150 mM NaCl). Lithium dodecyl sulfate (LDS) sample buffer (Invitrogen) was added to 20 μg of lysate and 20 μl of IP samples. Samples were separated by SDS-PAGE, transferred to nitrocellulose membranes and probed with antibodies to Myc (clone 4A6 Millipore), Flag (Sigma) or calnexin (Abcam).

### 2.9 CEBPA overexpression and qPCR

The human CEBPA cDNA clone (Origene: RG218955) was subcloned into pLXI_TRC401 a lentiviral expression vector containing an inducible Tet-on TRE promoter.

SGBS cells were transduced with pLXI-CEBPA or pLXI-GFP as described previously and 0, 0.1 or 1 μg/ml doxycycline was added into the culturing media. Cells were collected 48 h after doxycycline induction. Subsequently total RNA was extracted (Qiagen #74181) and cDNA synthesized (ThermoFisher #K1691) according to the manufacturer's protocol. Quantitative PCR was performed on the cDNA using Taqman probes for CEBPA (ThermoFisher Hs00269972_s1), AGPAT2 (ThermoFisher Hs00944961_m1) and Cyclophilin (ThermoFisher 4326316E). Fold-changes in comparison to the 0 umg/ml were calculated using the 2^−ΔΔCT^ method.

### 2.10 Fluorescence in situ hybridization of AGPAT2 and FACS sorting of SGBS cells

For the putative CEBPA binding sites identified in the AGPAT2 first intron and promoter (S1, S2, W; Figure 4B), the genomic sequences containing 20 bps flanking either the CEBPA motifs or CEBPA ChIP-peaks (sizes 52–164 bp) were inputted into the sgRNA designer as with the gene ablation constructs and manually curated to select sgRNAs that had the cut sites within the putative binding sites with minimal off-target effects (Supplementary Table 3). These were cloned into lentiCRISPRv2 as described above along with a guide targeting a control region in the AGPAT2 first intron (Int1Control). Four pools of polyclonal lentivirus containing all the sgRNA constructs for S1, S2, W, or the Int1Control sites were produced. A fifth pool containing all the guides for all three putative binding loci (S1 + S2 + W) was also produced.

SGBS cells were transduced with virus pools (S1, S2, W, S1 + S2 + W, Int1Control) and differentiated as described above. After four days, the transduced, partially differentiated SGBS adipocytes were trypsin-detached, pelleted and washed with PBS to carry out the RNA fluorescence in situ hybridization for AGPAT2 gene expression (PrimeFlow^®^ RNA Assay, #88-18005) with the following modifications:

A. All spins were performed at 1000 g for 8 min.
B. Two permeabilization steps followed the fixation step, skipping the intracellular cell staining step.
C. The target Probe against AGPAT2 (customized high-sensitivity AGPAT2 Type1 PrimerFlow probe) was used for hybridization.
D. After the PreAmp, Amp and Label Probe hybridizations and washes, cells were brought to 2 × 10^6^/ml with PrimeFlow RNA Storage Buffer or Flow Cytometry Staining Buffer, filtered with 100 μm cell strainer and stored at 4 °C

FACS sorting was performed on a SONY SH800S cell sorter with 100 μm chip.

At least 70,000 cells were sorted into 100 μl 2× lysis buffer (2% SDS, 20 mM EDTA, 100 mM Tris–HCl, pH 8.1) and adjusted to 1× lysis buffer by either 2× lysis buffer or 1× PBS depending on the collected volume. Samples were incubated at 65 °C for 10 min, cooled to 37 °C and mixed with RNase Cocktail (Thermo Fisher, AM2286, 1:50) and incubated for a further 30 min at 37 °C. Then samples were mixed with Proteinase K (NEB, P8107S, 1:10) and incubated at 37 °C for 2 h and inactivated at 95 °C for 20 min. Genomic DNA was extracted using SPRI beads (Agencourt AMPure XP beads, Cat. No. A63881). A 160 bp amplicon encompassing the S1, S2, or Int1Control sites was generated and shotgun sequenced (paired-end 500 cycle) on an Illumina Miseq using standard protocols (https://portals.broadinstitute.org/gpp/public/resources/protocols).

Genomic DNA modification at each locus (each distinct allele and the number of reads containing that allele) was tabulated using CrisprVariants with default parameters [17]. Disruptive indels were quantified by aggregating the number of reads >3 base pair indels in each sample (S1, S2, Int1C) in the AGPAT2 high and low fractions. For each site an odds ratio ([disruptive_low_/non-disruptive_low_]/[disruptive_high_/non-disruptive_high_]) was calculated with 95% confidence intervals using logistic regression as instantiated in R3.42.

## 3 Results

### 3.1 Gene selection, ablation in adipocytes and imaging

Genes for profiling in adipocytes were selected from genetic association studies for body mass index (BMI) [12], Waist-to-hip ratio adjusted for BMI (WHRadjBMI), fasting insulin (Fins) [25] and type 2 diabetes (T2D) [26], [27], [28] (Figure 1A). Loci from these studies were considered if they were associated with a *p*-value of 5 × 10^−8^ (i.e. the threshold for genome-wide significance) or less. A genomic window of 250 kilobases surrounding the lead SNP at each locus was scanned for protein coding genes. The resulting list of 1180 genes was filtered by adipocyte expression having to meet or exceed a threshold of 0.1FPKM during any sampled timepoint (day 0, 4, 8, 15) of SGBS adipocyte differentiation. The list was further narrowed down by removing genes at loci for which an alternate causal transcript is widely accepted, resulting in 84 genes. An additional 41 genes associated with Mendelian forms of metabolic disease such as congenital lipodystrophy, insulin resistance and known regulators of adipocyte differentiation and insulin signaling were selected. Another eight genes known to be cell essential regulators were selected to create the final list of 133 genes (Supplementary Table 1).

Three independent CRISPR/CAS9 targeting constructs (lentiCRISPRv2 vector) were designed for each of the 133 genes using “Ruleset 2” as described previously [15]. These were introduced by lentiviral transduction into SGBS pre-adipocytes (Figure 1A), a diploid, human cell model for adipocytes [18]. After a ten day incubation, the lentivirally targeted pre-adipocytes were differentiated with a standard cocktail of adipogenic inducers for 12 days, fixed, stained and imaged by high-content microscopy, generating ∼77,000 images. An unbiased selection of 425 morphologic features (such as number of lipid droplets, size, etc.) was quantified from each image (Figure 1B). This set of features was filtered by reproducibility among biological replicates (*n* = 28 features removed) and further reduced to set aside redundant (i.e. highly correlated) features across all guides (*n* = 249) to generate a set of 148 independent core features (Supplementary Table 2). The values for the core independent features were averaged across all three targeting constructs for each gene to generate a “morphologic profile” for every gene.

### 3.2 Identification of morphologic interactions and validation of gene modification

**Figure 2:**
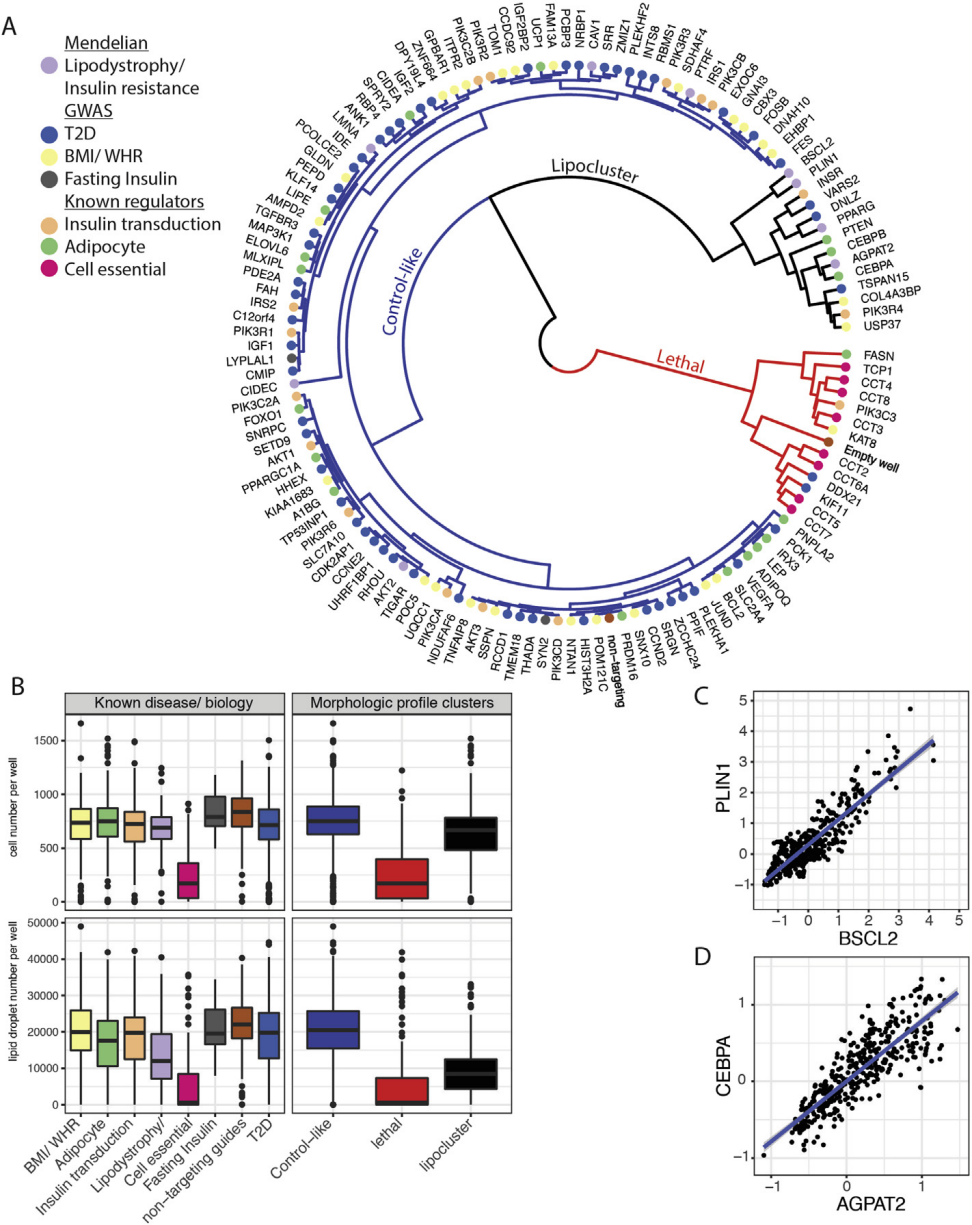
**Comparison of morphologic profile annotations to known biology and identification of novel morphologic interactions.** (A) Morphologic correlations overlaid with known gene annotations. Shown is a circular dendrogram relating the morphologic profiles obtained from perturbing 133 genes, non-targeting controls, and empty wells. The distal leaves of the dendrogram (individual genes) are labeled and color-coded according to their relation to known genetic disease associations and cellular biology. The first three major branches of the dendrogram are highlighted in color and named “control-like” (blue; inclusion of non-targeting controls), “lethal” (red; inclusion of empty wells) and “lipocluster” (black; inclusion of many known lipodystrophy genes). (B) Cell number and lipid accumulation in sets of genes according to known disease/biological associations or morphologic similarity. Boxplots show median, 25th and 75th percentiles of cell number (top panels) or lipid droplet number (bottom panels) in the 133 gene knockouts grouped by known disease/biological associations (left panels) or by morphologic profile similarity (as shown in 2A). (C) Scatterplot comparing 425 morphologic features (*Z*-scores) identified from human adipocytes ablated for BSCL2 or PLIN1. Pearson *r* = 0.89, *p* < 2.2 × 10^−16^. (D) Scatterplot comparing 425 morphologic features (*Z*-scores) identified from human adipocytes ablated for CEBPA or AGPAT2. Pearson *r* = 0.85, *p* < 2.2 × 10^−16^.

To investigate potential functional relationships between genes, we performed unsupervised clustering of the morphologic profiles (*n* = 148 core features) for all 133 genes and included the morphologic profiles of control cells infected with constructs targeting no genomic sequences (non-targeting) and cells exposed to lethal antibiotic selection (empty wells) (Figure 1B and Figure 2A). Three major clusters of morphologic similarity emerged. The largest cluster (“control-like”) consisted of 106 genes and non-targeting constructs. A second cluster (“lethal”) contained 13 genes, and was enriched for known cell essential genes (Figure 2A; odds-ratio = 29, hypergeometric *p* < 10^−9^ of essential genes in “lethal” vs other clusters). The third cluster (“lipocluster”) consisted of 14 genes and was enriched for genes known to cause lipodystrophy and syndromic insulin resistance (odds-ratio = 8.8, hypergeometric *p* < 0.008 of lipodystrophy genes in “lipocluster” vs other clusters).

To evaluate biological plausibility in the relationships identified *ab initio* by morphologic profile clustering, we examined two individual features, cell number and lipid droplet number, that are known to correspond with the extent of adipocyte differentiation [16] with respect to known disease/functional annotations of the underlying genes (Figure 2B). As expected, ablation of cell essential genes decreased both cell number and lipid droplet accumulation (Figure 2B left panels). A similar pattern was observed in the “lethal” cluster defined *ab initio* (Figure 2B right panels). Ablation of lipodystrophy genes decreased lipid droplet number without severely decreasing cell number (Figure 2B). A similar pattern was identified in the “lipocluster” with large decreases in lipid droplet accumulation without large decreases in cell number compared to the “control-like” cluster. Thus genes clustering in the lipocluster represent genes that, when ablated, produce morphologic changes that phenocopy genes known to cause syndromic human lipodystrophy and insulin resistance.

To further confirm that the cellular phenotypes observed in the lipocluster were due to the ablation of the targeted genes we quantified the gene modification efficiency of the CRISPR/CAS9 targeting constructs. SGBS pre-adipocytes were transduced with the corresponding targeting constructs, incubated for ten days, and then sequenced at the genomic DNA surrounding the targeting site in at least 5000 cells per targeting construct. The gene modification efficiency (i.e. resulted in insertions/deletions) overall was high, ranging from 72 to 98 percent of sample alleles for the most effective targeting guide per gene (Supplementary Table 1).

The lipocluster contained five genes DNLZ, VARS2, COL4A3BP, USP37, and TSPAN15 not previously known to have a role in adipocyte biology or function. Three genes (DNLZ, VAR2, and TSPAN15) were identified in T2D associated loci [26], [27], [28] and two (COL4A3BP and USP37) from BMI associated loci [12], [13] (Figure 2A). The proteins encoded by these genes ranged in function from enzymes (VARS2: valyl-tRNA synthetase [29], [30], USP37: deubiquitinase [29]) to chaperones (DNLZ [31], COL4A3BP [32]) to a transmembrane signaling protein (TSPAN15 [33]). With regard to human genetic variation in these genes, each contained dozens of rare missense variants of unknown functional consequence when queried in exome sequencing datasets [34], [35].

We further investigated genes in the lipocluster for novel mechanistic associations using their underlying morphologic profiles. To maximize biologically meaningful clustering we first evaluated the stability of clustering using resampling techniques in order to identify pairs of genes that co-cluster robustly. Two such pairs of genes were identified in the lipocluster, BSCL2 and PLIN1 (resampling *p* = 0) and CEBPA and AGPAT2 (resampling *p* = 0.01) (Supplementary Figure 1). Examination of all the extracted morphologic features in these gene pairs demonstrated similar perturbed values across multiple features as evidenced by strong linear correlation (Pearson *r* = 0.89, *p* < 2.2 × 10^−16^) BSCL2-PLIN1 Figure 2C, *r* = 0.85, *p* < 2.2 × 10^−16^ CEBPA-AGPAT2 Figure 2D).

### 3.3 Validation of protein–protein interaction between BSCL2 and PLIN1

**Figure 3:**
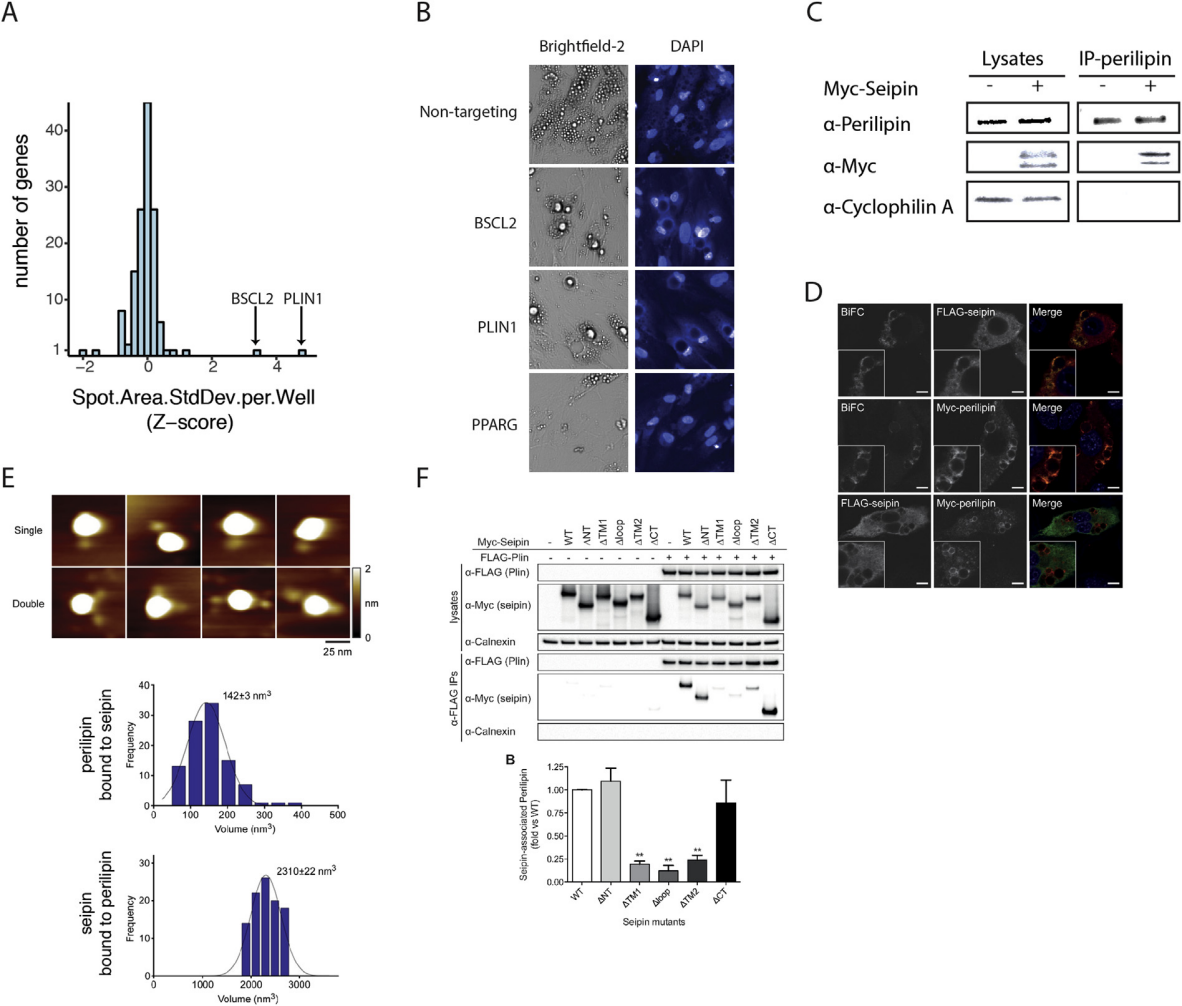
**Protein–protein interaction of BSCL2 and PLIN1 at the lipid droplet surface.** (A) Histogram of *Z*-scores for variation in lipid droplet size in SGBS adipocytes ablated for 133 genes. Variation in lipid droplet size is quantified by automated image analysis and feature extraction and summarized as the standard deviation for all cells exposed to targeting constructs for a gene. Adipocytes targeted with constructs against BSCL2 or PLIN1 result in the most extreme *Z*-scores for variation in lipid droplet size among the 133 genes. (B) 20× brightfield and fluorescence (488 nm, DAPI stained) images of SGBS cells exposed to gene targeting and non-targeting CRISPR/CAS9 constructs and subsequently differentiated. Residual large lipid droplets are observed with BSCL2 or PLIN1 ablation when compared to control or PPARG ablation. (C) Co-immunoprecipitation and western blotting of seipin and perilipin in human adipocytes. SGBS adipocytes were transduced with Myc-seipin and differentiated for seven days. Endogenous perilipin was immunoprecipitated and co-immunoprecipitation of exogenous Myc-seipin is observed. Cyclophilin A was blotted as a cell loading control. (D) 3T3-L1 cells were induced to differentiate and co-transfected with FLAG-seipin-YFPn and Myc-perilipin-YFPc on day 2 of differentiation. Following a temperature shift to induce the formation of reconstituted YFP, cells were fixed on day 5 of differentiation and stained for FLAG-seipin (top panels) or Myc-perilipin (middle panels) and DAPI to label nuclei. The direct interaction between seipin and perilipin is indicated by the presence of YFP signal. Identically transfected cells were used to co-immunostain for FLAG-seipin-YFPn and Myc-perilipin-YFPc (bottom panels). Note that these cells were not temperature shifted to prevent formation of YFP, which would confound the Alexa Fluor 488 fluorescence used to detect the FLAG epitope. Top and middle panels, individual images are shown in grayscale and merged images show overlay of YFP (yellow) and FLAG-seipin or Myc-perilipin (red). Bottom panels, individual images are shown in grayscale and merged image shows overlay FLAG-seipin (green) and Myc-perilipin (red), inset boxes show zoomed images. Scale bars, 10 μm. (E) AFM analysis of the interaction of perilipin with seipin. FLAG-perilipin and seipin-Myc were co-expressed in tsA 201 cells and proteins were isolated using anti-Myc immunoaffinity chromatography. AFM images of isolated seipin dodecamers (large particles) singly (upper panels) or doubly (lower panels) decorated by smaller perilipin particles. Scale bar, 25 nm; height scale, 0–2 nm. Histograms show the frequency distribution of volumes of seipin particles bound to perilipin (*n* = 100) and of perilipin particles bound to seipin particles (*n* = 100). The curve indicates the fitted Gaussian function. The peak of the distribution (±SEM) is indicated. (F) HEK293 cells were transfected with FLAG-perilipin in the absence or presence of either wild-type Myc-seipin (WT) or mutants lacking the N terminus (ΔNT), first transmembrane domain (ΔTM1), ER luminal loop region (Δloop), second transmembrane domain (ΔTM2), or the C terminus (ΔCT). Lysates or anti-FLAG immunoprecipitates were immunoblotted for FLAG, Myc and the ER membrane protein calnexin. Quantification of the interaction of mutant vs. wild-type seipin from replicate experiments is shown in the lower panel. Data are means ± SEM (*n* = 3). ** indicates *p* < 0.01 versus co-immunoprecipitation with wild-type seipin.

To direct further mechanistic investigation, we initially focused on individually striking features that were similarly perturbed by both gene knockouts. BSCL2 and PLIN1 ablated cells demonstrated several cellular features ranking in the top 1 percent among all gene perturbations (i.e. *Z*-score > 2.5) (Supplementary Table 2). All of these high ranking features related to lipid droplet morphology. In the most extreme feature, variation in lipid droplet size, PLIN1 ranked first (PLIN1 *Z*-score = 4.7) and BSCL2 second (BSCL2 *Z*-score = 3.8) among all genes perturbed (Figure 3A). Examination of the raw images of BSCL2/PLIN1 ablated cells (Figure 3B) revealed cells with decreased lipid accumulation overall, but with strikingly large residual lipid droplets as compared to control cells or cells ablated for other lipodystrophy genes such as PPARG.

These observations led us to hypothesize that seipin and perilipin, the gene products of BSCL2 and PLIN1 respectively, function together in lipid droplet biology. To test this hypothesis, we first assessed if seipin and perilipin interact physically using co-immunoprecipitation and Western blot in differentiating SGBS cells. Immunoprecipitation of endogenous perilipin in differentiated cells co-immunoprecipitated myc-tagged seipin (Figure 3C). In order to specifically localize the site of this interaction, we performed bimolecular fluorescence complementation (BiFC) analyses. When perilipin-YFPc and seipin-YFPn constructs were expressed in 3T3-L1 adipocytes, an interaction was noted at the junction of the lipid droplet surface and the endoplasmic reticulum (Figure 3D). To evaluate if the interaction between seipin and perilipin occurs via direct association of the two proteins, we employed atomic force microscopy (Figure 3E). This revealed large particles consistent with the molecular volume previously reported for seipin dodecamers (2310 ± 22 nm^3^; *n* = 100) [22], [24]. Associated with these were smaller particles with molecular volume appropriate for perilipin (142 ± 3 nm^3^; *n* = 100). Overall, 24% of immunoprecipitated seipin particles (89/365) were decorated with perilipin particles. While almost all decorated seipin dodacamers were associated with one perilipin molecule, we occasionally noted two perilipin particles per seipin dodecamer. These experiments revealed that dodecamers of seipin can bind directly to perilipin. Finally, we examined which regions of seipin are responsible for the interaction with perilipin by co-immunoprecipitation with mutated forms of seipin lacking key functional domains (Figure 3F). The first and second transmembrane domains of seipin as well as its ER luminal loop domain were necessary for interaction with perilipin, but the N and C terminal domains were dispensable. Seipin has been reported to localize to lipid droplet ER junctions and to particularly influence a key step in the maturation of lipid droplets [36]. We propose that the direct interaction we have identified with seipin may facilitate the recruitment of perilipin 1 to these maturing lipid droplets. Seipin has been reported to interact with a variety of proteins that mediate lipid droplet biogenesis [37] and directly to phospholipids on the droplet surface [38], [39]. Supporting our findings are an evolutionarily conserved functional interaction of the yeast orthologue of seipin with a novel yeast perilipin-like protein Pet10p [40], and a reported interaction between seipin and the lipid droplet protein ADRP/Plin2 [41]. In summary, our morphologic profiling method uncovered a novel direct protein–protein interaction between perilipin and seipin, which occurs at the lipid droplet surface in differentiating adipocytes.

### 3.4 Validation of genetic interaction between CEBPA and AGPAT2

**Figure 4:**
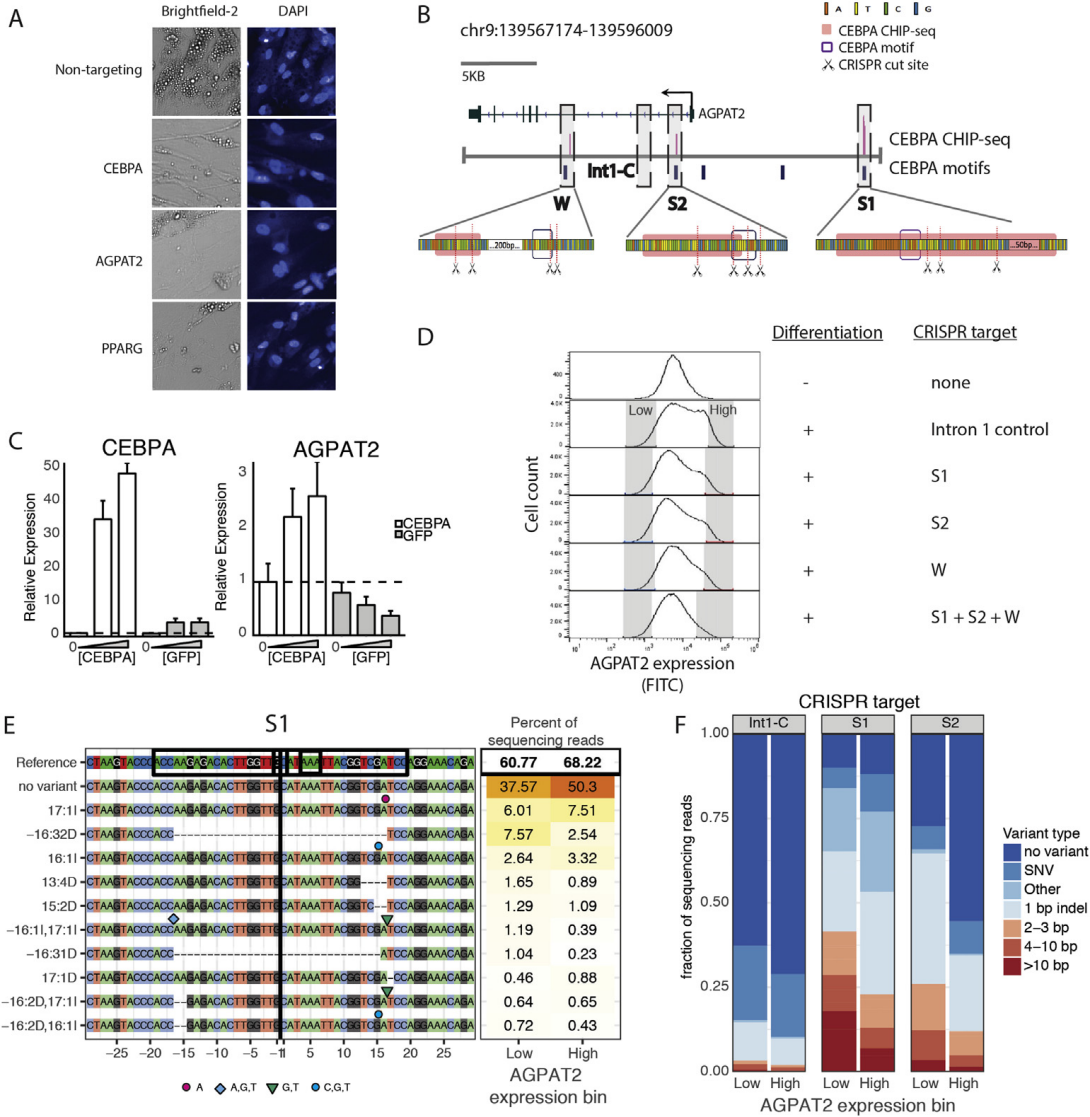
**Genetic interaction between AGPAT2 and CEBPA.** (A) 20× brightfield and fluorescence (488 nm, DAPI stained) images of SGBS cells exposed to gene targeting and non-targeting CRISPR/CAS9 constructs and subsequently differentiated. Decreased lipid accumulation is observed with AGPAT2, CEBPA, or PPARG ablation. (B) (upper) The AGPAT2 gene structure is shown (hg19) with overlaid tracks showing the genomic positions of C/EBPa ChIP-Seq peaks in differentiated SGBS cells (Galhardo et al., 2014). The genomic position of high-scoring C/EBPa consensus motifs is also shown alongside a selected control region in the first intron (Int1C). (lower) Expanded base pair resolution view of three putative C/EBPa binding sites identified on the basis of overlapping of CHIP-seq (pink boxes) and consensus motif (blue boxes) data. For each site identified site S1, S2 and W the positions of the predicted cut site of selected CRISPR/CAS9 constructs is shown. (C) Dosed overexpression of *CEBPA* in undifferentiated SGBS cells using a doxycyline-inducible transgene with assessment of AGPAT2 gene expression. RT-qPCR fold-change data are shown demonstrating a dose response for AGPAT2 induction in response to CEBPA over-expression as compared to GFP overexpression. (D) FACS sorting of differentiating SGBS cells according to AGPAT2 gene expression. SGBS cells were treated with CRISPR constructs targeting putative C/EBPa binding sites or a control region in the first intron, differentiated, and stained for AGPAT2 expression. Histograms of AGPAT2 gene expression are shown for CRISPR-treated populations as well as undifferentiated cells. In differentiated cells a second peak of increased AGPAT2 gene expression is observed and is attenuated by treatment of CRISPR constructs targeting C/EBPa binding sites. Grey boxes denote the sorting gates (comprising 6.5–8.5% of the total distribution) of low and high AGPAT2 expressing cells collected for genomic analysis. (E) Indel quantification at the S1 C/EBPa binding site of SGBS adipocytes sorted by AGPAT2 expression. SGBS cells were treated with S1 site targeting CRISPR constructs as shown in D) with 70,000 cells shotgun from the low and high ends of the AGPAT2 expression distribution. Listed are the ten most frequent alleles collectively comprising 60 and 68 percent of all sequenced alleles. Large indels are enriched in treated cells expressing low levels of AGPAT2. (F) Indel quantification at the intron 1 control, S1, or S2 C/EBPa binding sites in SGBS adipocytes sorted by AGPAT2 expression. SGBS cells were treated with control, S1, or S2 site targeting CRISPR constructs as shown in D) with 70,000 cells shotgun from the low and high ends of the AGPAT2 expression distribution. The stacked barplots show the relative frequency and type of allele at the sequenced site corresponding to each targeting construct. An increase in proportion of large indels can be seen in the low AGPAT2 expression bin in adipocytes treated with S1 or S2 targeting constructs.

In contrast to the extreme features observed after BSCL2-PLIN1 ablation, visual examination of the individual features underlying the morphologic correlation between CEBPA-AGPAT2 did not reveal any obviously perturbed features. However, the morphologic correlation across all features taken together was robust (*r* = 0.85, *p* < 2.2 × 10^−16^) suggesting that many features are similarly perturbed by CEBPA/AGPAT2 ablation, but no individual feature is strikingly perturbed for either gene (*Z*-score_max_ CEBPA = 1.3 and *Z*-score_max_ AGPAT2 = 1.5, Figure 2D, Supplementary Table 2). Examination of the raw images of CEBPA/AGPAT2 ablated cells revealed cells with decreased lipid accumulation compared to controls, but did not appear distinctive on manual visual inspection in comparison to ablation of other lipodystrophy genes such as PPARG (Figure 4A). Knowing that CEBPA is a transcription factor that promotes adipocyte differentiation [42] and that AGPAT2 encodes an acyltransferase enzyme required for adipocyte differentiation [43], we hypothesized a genetic regulatory interaction with CEBPA regulating the transcription of AGPAT2. In order to substantiate this hypothesis, we first examined the genomic DNA at the *AGPAT2* locus for potential C/EBPa binding sites. We scanned a genomic window ±20 kilobases (kb) surrounding the AGPAT2 gene for sequences likely to be C/EBPa consensus motifs and overlaid previously published C/EBPa binding (ChIP-seq) data collected from differentiated SGBS cells [44] (Figure 4B). Three potential binding sites were identified – Strong 1 (S1): 11 kb upstream of the AGPAT2 exon start, Strong 2 (S2): 0.8 kb within the first intron and Weak (W): 7.6 kb in the first intron. The three sites were labeled “strong” (S) and “weak” (W) on the strength of the evidence for being a C/EBPa binding site; S1 and S2 had ChIP-seq peaks that overlapped a C/EBPa consensus binding motifs whereas W had a ChIP-seq peak that did not overlap the most proximate consensus binding motif. To test for a functional regulatory interaction we over-expressed CEBPA in undifferentiated SGBS cells and found this to be sufficient to induce *AGPAT2* gene expression (Figure 4C). To further assess if this functional interaction was direct and required the identified C/EBPa binding sites (Figure 4B), we utilized genome engineering to selectively disrupt these sites in differentiating SGBS cells and examined the transcriptional effect on AGPAT2. We treated undifferentiated SGBS cells with an array of CRISPR/CAS9 constructs targeting the S1, S2 and W sites singly and in combination, then applied a differentiation stimulus and examined AGPAT2 gene expression by fluorescence in situ hybridization and flow cytometry (FISH-FLOW) (Figure 4D). In undifferentiated pre-adipocytes a single peak of low AGPAT2 expression is observable whereas differentiating cells treated with a control construct targeted to the AGPAT2 first intron exhibit a second peak of AGPAT2 expression with ∼10-fold increased staining intensity. This second peak of increased AGPAT2 expression is attenuated in differentiating adipocytes treated with constructs targeted to the S1, S2, or W sites. The second peak is almost fully attenuated in cells treated with constructs targeting all three sites simultaneously. Given that our genome engineering strategy leveraged CRISPR/CAS9 double strand genomic DNA breaks and error prone non-homologous end joining repair [45] we expected that the resulting cell populations likely contained a spectrum of DNA alterations ranging from perfect repairs and single nucleotide changes to large insertions/deletions (indels). We utilized this experimentally induced genetic variation to further assess the requirement for the C/EBPa binding sites in AGPAT2 gene expression predicting that cells from the low end of the distribution would harbor more disruptive indels compared to cells from the high end of the distribution (Figure 4E). This prediction was tested by collecting 70,000 cells via FACS from the low and high ends of the AGPAT2 expression distribution in the engineered cell populations and sequencing their DNA at the corresponding targeting sites (S1 or S2). We examined S1 and S2 because the compact size of these binding sites enabled them to be fully encompassed in DNA fragments amenable for shotgun sequencing. Among the most frequently occurring mutations, large deletions were observed more frequently in cells from the bottom of the AGPAT2 gene expression distribution vs the top (Figure 4E alleles −16:32D, −16:31D targeted at S1). Systematic quantification of all of the sequenced DNA demonstrated an accumulation of disruptive indels (i.e. >3 bp) in cells with low versus high AGPAT2 expression the populations targeted at S1 (odds ratio: 2.67, 95% CI 2.64–2.70) or S2 (odds ratio: 2.70, 95% CI 2.66–2.74) as compared to control (odds ratio: 1.73, 95% CI 1.60–1.88) (Figure 4F).

## 4 Discussion

In this study we developed an approach for functional characterization of human disease genes by combining systematic genetic perturbation in a native cellular context with in depth quantification of the resulting cellular morphology. We tested this approach with metabolic disease genes expressed in adipocytes, identifying and subsequently validating novel interactions between BSCL2 and PLIN1 (protein–protein) as well as CEBPA and AGPAT2 (genetic regulatory). These results demonstrate that our assay system can interrogate multiple cellular mechanisms revealing new biology regarding the two most frequently identified gene disruptions (BSCL2 and AGPAT2) [46] leading to congenital generalized lipodystrophy. Specifically CEBPA was found to regulate AGPAT2 expression and perilipin was identified as a novel seipin interacting protein.

Our approach is likely to generalize to other genetic diseases with cellular origins. In its technical aspects our use of CRISPR/CAS9 allows a high degree of gene targeting specificity [47] and enables any mammalian cell type to be targeted [48]. Our stably integrating lentiviral vector system drives gene disruption towards completion [49] enabling high confidence modification (Figure 1B, Supplementary Table 1). The use of cellular morphology as a readout for annotating gene function is a relatively recent advance [50], and has thus far been deployed in cancer cell lines (e.g., U2OS [8], A549 [51], HeLa [6], [7]). Successful examples of targeted use for chemical screens in patient derived cells (e.g., iPS [52] and vascular endothelial cells [53]) suggest that our study in human adipocytes will likely translate to other disease relevant cell models.

This study highlights the use of computer vision to identify morphologic correlations not evident from human visual inspection of microscopy images. The morphologic consequence of ablating either BSCL2 or PLIN1 in differentiating adipocytes (overall decreased lipid accumulation and unusually large lipid droplets; Figure 3A) could have been detected by human visual inspection of ∼77,000 images, but the CEBPA-AGPAT2 interaction could not have been distinguished manually (Figure 4A). This is likely due to the lack of “processing” performed by computer vision versus human vision. In computer vision, the image analysis software retains the “raw data” consisting of quantified, individual morphological feature whereas in human vision these features are integrated by the visual cortex into a synthesized representation of the image from which the raw features are not easily discernible. It is this ability to quantify hundreds of individual features by computer vision that enables robust correlations to be identified even when any individual feature does not stand out.

As a proof-of-concept, the findings of our study are subject to a number of limitations. Notably, while many previously known lipodystrophy genes fell within the “lipocluster” (Figure 2A), several did not (e.g., CAV1, PTRF, AKT2, CIDEC, LMNA). In the case of genes causing generalized lipodystrophy, this may reflect the fact that while disruption of BSCL2 or AGPAT2 leads principally to a lack of adipose tissue, PTRF and CAV1 mutations cause a more complex phenotype in affected patients [46]. The majority of mutations causing partial lipodystrophy exhibit autosomal dominant inheritance and may act as dominant negative forms or have complex and poorly understood molecular actions, as in the case of LMNA [46], [54]. Hence the actions of these pathogenic mutations might not be easily modeled by loss-of-function or ablation, as in our experiments. Furthermore, our results should also not be interpreted as comprehensive, i.e., any particular gene falling in the “control-like” cluster does not indicate a lack of relevance to disease or to adipocyte biology. For example, leptin ablation would not be expected to read out in our cell-autonomous assay. Also our experiments were performed under standard differentiation conditions that include a pharmacological dose of rosiglitazone, a PPARg inducer not found in nature [55]. Thus, a gene impairing adipocyte differentiation *in vivo* more subtly might be masked by this stimulus *in vitro*. By deleting genes prior to differentiation our study cannot distinguish between morphologic effects secondary to changes in differentiation versus mature adipocyte function. Overall, the novel findings regarding the regulation of AGPAT2 and function of seipin demonstrate the power of our morphometric approach to identify new mechanistic insights regarding genes important for human metabolic disease.

## 5 Conclusions

In the future, our approach may be applied to any disease-cell model pair, incorporating multiple different cellular stimuli with the same readout to tease out more subtle genetic effects and highlight other morphologic interactions. Incorporating different cellular stimuli would also enable higher order clusters with multiple genes to be linked to cellular pathways, as genes that are critically dependent on one another will be more likely to cluster under multiple conditions (e.g. known CCT chaperonin complex genes co-clustering; Figure 2A). Finally, by virtue of using human cell models and a multi-dimensional morphometric readout, our approach provides an assay system to functionally study human coding variants in the ablated genes. As we have previously exemplified with PPARG [16], [49] the ability to experimentally determine the function of rare coding variants can establish genotype–function–phenotype correlations for use in clinical diagnosis of novel variants in a known disease gene. Applied to novel genes such as the ones identified in this study with no previous link to adipocyte function (DNLZ, VARS2, COL4A3BP, USP37, and TSPAN15), transgene complementation with human coding variants reading out “rescue” of the identified morphologic profile with correlation to metabolic disease phenotypes would provide strong evidence for disease causation and direction of effect.

## Author contributions

EDR and ARM designed and directed the study

XZ, RR, AIP, DFR, SG, VW, MTS and JGD designed and executed the screening

RR, MKA, KH and ARM analyzed and interpreted the high-content imaging data

MFMS, MMUT, JME and JJR designed and performed the validation studies for BSCL2-PLIN1

YJ, UA and AB performed the validation studies for CEBPA-AGPAT2

JF and ARM analyzed the CRISPR variant data

YJ, MFMS and ARM made the figures and wrote the manuscript

All authors read, edited and approved the final manuscript

## Figures and Tables

**Figure:**
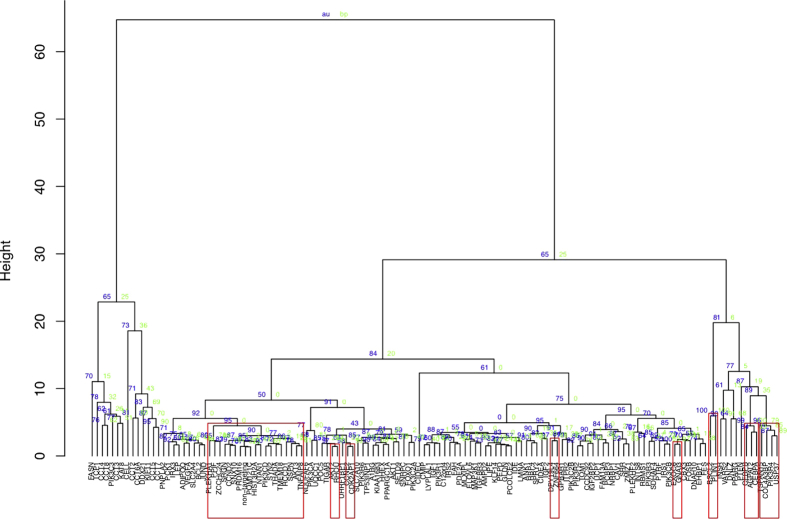
figs1
