# Supplementary material for: Discovering metabolic disease gene interactions by correlated effects on cellular morphology
Source: Mol Metab. 2019 Mar 13;24:108–19. doi: 10.1016/j.molmet.2019.03.001 (PMC6531784; doi:10.1016/j.molmet.2019.03.001)
Supplement: Multimedia component 3 [file mmc3.pdf]

CRISPR targeting constructs

C/EBPa site	targeting specifying sequence for CRISPR (sgRNA)	Orientation
S1-1	GCATAAATTACGGTCGATCCAGG	Reverse
S1-2	GCAACCAAGTGTCTCTTGGTGGG	Forward
S1-3	TTGCATAATGTTCTGTTTCCTGG	Forward
S2-1	CCAGCCAGTGGCTTTTGCAATGG	Forward
S2-2	CAGGCCATTGCAAAAGCCACTGG	Reverse
S2-3	CCATTGCAAAAGCCACTGGCTGG	Reverse
W-1	CGCACACTCAGGGGAACTGCAGG	Reverse
W-2	TGGTAGCACCCGCACACTCAGGG	Reverse
W-3	AGCACCACCTCAGGCTGATGGGG	Reverse
W-4	AGCATTGCCCCATCAGCCTGAGG	Forward
Intron1C	CAAGGCGGGATGGGTCCATAGGG	Reverse

Genomic DNA sequencing primers

C/EBPa site	Forward primer	Reverse primer
S1	ccatctcatccctgcgtgtctccgcaaggctccgtctcaaaa	cctctctatgggcagtcggtgatgagccagttgtgcaaacagtg
S2	ccatctcatccctgcgtgtctccccctctccctgttagccg	cctctctatgggcagtcggtgatgcactgcccctggaaccac
W-ChIP peak	ccatctcatccctgcgtgtctccgctggattgcgcaaatgagt	cctctctatgggcagtcggtgatgatctgccttcctcagccaac
W-motif	ccatctcatccctgcgtgtctcccccttgactcagtttcccc	cctctctatgggcagtcggtgatggttcccatgcgacacaaacc
Intron1C	ccatctcatccctgcgtgtctcccctgcactcctacacatccg	cctctctatgggcagtcggtgatgctggtgttttccgacaagtgt
